#  Eye and head movements while looking at rotated scenes in VR. Session "Beyond the screen's edge" at the 20th European Conference on Eye Movement Research (ECEM) in Alicante, 19.8.2019 

**DOI:** 10.16910/jemr.12.7.11

**Published:** 2019-11-25

**Authors:** Nicola C. Anderson, Walter F. Bischof

**Affiliations:** University of British Columbia, Vancouver; SCIANS Ltd,Iffwil (Switzerland) and University of British Columbia, Vancouver

**Keywords:** virtual reality, eye movements, head movements

## Abstract

We examined the extent to which image shape (square vs. circle), image rotation, and image content (landscapes vs. fractal images) influenced eye and head movements. Both the eyes and head were tracked while observers looked at natural scenes in a virtual reality (VR) environment. In line with previous work, we found a horizontal bias in saccade directions, but this was affected by both the image shape and its content. Interestingly, when viewing landscapes (but not fractals), observers rotated their head in line with the image rotation, presumably to make saccades in cardinal, rather than oblique, directions. We discuss our findings in relation to current theories on eye movement control, and how insights from VR might inform traditional eyetracking studies. - Part 2: Observers looked at panoramic, 360 degree scenes using VR goggles while eye and head movements were tracked. Fixations were determined using IDT (Salvucci & Goldberg, 2000) adapted to a spherical coordinate system. We then analyzed a) the spatial distribution of fixations and the distribution of saccade directions, b) the spatial distribution of head positions and the distribution of head movements, and c) the relation between gaze and head movements. We found that, for landscape scenes, gaze and head best fit the allocentric frame defined by the scene horizon, especially when taking head tilt (i.e., head rotation around the view axis) into account. For fractal scenes, which are isotropic on average, the bias toward a body-centric frame gaze is weak for gaze and strong for the head. Furthermore, our data show that eye and head movements are closely linked in space and time in stereotypical ways, with volitional eye movements predominantly leading the head. We discuss our results in terms of models of visual exploratory behavior in panoramic scenes, both in virtual and real environments.

**Video stream:** https://vimeo.com/356859979

**Production and publication of the video stream was sponsored by SCIANS Ltd** http://www.scians.ch/

